# Supplementary material for: A Likelihood Approach for Real-Time Calibration of Stochastic Compartmental Epidemic Models
Source: PLoS Comput Biol. 2017 Jan 17;13(1):e1005257. doi: 10.1371/journal.pcbi.1005257 (PMC5240920; doi:10.1371/journal.pcbi.1005257)
Supplement: S1 File — (TAR.GZ) [file pcbi.1005257.s014.tar.gz › HSPH_Online-SI-Revision/IPoi09/n10-mild/n10-mild_BENCH_table0.pdf]

1	R0	{1.25046, 6.80278}	{1.7017, 11.7764}	{{1.68954, 11.6019}, {1.72065, 11.7288}}	{36.0859, 73.111}	inf	157.	805.471	{360., 1042.}	415.936	0.688608	newly	174.	477.706	{246., 607.}	178.645	0.438602	Reff	1.06127	1.09709	{0.8149, 1.50876}	20.4566	ar	3516.	6886.06	{6664., 7097.}	95.8492
2	R0	{1.32013, 4.45847}	{2.12421, 12.4436}	{{2.0968, 12.1899}, {2.21449, 13.2892}}	{60.9094, 179.1}	inf	321.	1430.21	{829., 1804.}	347.575	0.635243	newly	488.	807.946	{570., 995.}	70.5174	0.235143	Reff	0.980989	1.1521	{0.695928, 1.72546}	34.6711	ar	4420.	8287.3	{8121., 8490.}	87.4956
3	R0	{1.31785, 5.36796}	{1.78724, 6.63253}	{{1.78724, 6.63253}, {1.78724, 6.63253}}	{35.6179, 23.5577}	inf	363.	810.878	{575., 1067.}	123.893	0.334942	newly	488.	749.171	{489., 1115.}	58.2267	0.187423	Reff	1.00407	0.750292	{0.577815, 0.915603}	28.3687	ar	4942.	7262.24	{7098., 7489.}	46.9495
4	R0	{1.38801, 6.16031}	{2.33209, 16.8026}	{{2.22867, 15.6411}, {2.46477, 18.9032}}	{68.0169, 172.755}	inf	457.	1815.77	{1334., 2195.}	302.384	0.614477	newly	527.	756.842	{614., 878.}	50.3246	0.201278	Reff	1.01047	1.14476	{0.666967, 1.63118}	31.8907	ar	4790.	8685.63	{8459., 8859.}	81.3284
5	R0	{1.27955, 11.57}	{1.5973, 19.0861}	{{1.59433, 18.9003}, {1.59945, 19.1171}}	{24.8327, 64.9618}	inf	179.	662.178	{455., 861.}	276.189	0.567625	newly	120.	239.422	{170., 312.}	108.667	0.330159	Reff	1.07866	0.953576	{0.734148, 1.25139}	20.562	ar	3460.	6420.13	{6210., 6607.}	85.553
6	R0	{1.22184, 4.34395}	{1.47488, 4.06661}	{{1.47488, 4.06661}, {1.47488, 4.06661}}	{20.7097, 6.38451}	inf	231.	314.407	{148., 525.}	54.2569	0.190278	newly	353.	459.778	{188., 844.}	54.4224	0.198238	Reff	1.01352	0.784024	{0.670923, 0.943923}	24.2861	ar	3524.	5618.85	{5367., 5977.}	59.4453
7	R0	{1.23856, 4.59068}	{1.87268, 9.18983}	{{1.87476, 9.18466}, {1.87476, 9.18466}}	{51.1984, 100.184}	inf	200.	1059.36	{781., 1344.}	429.678	0.713098	newly	288.	765.356	{522., 1020.}	166.304	0.410032	Reff	0.970659	0.864068	{0.600861, 1.21394}	23.3744	ar	3855.	7604.33	{7502., 7761.}	97.259
8	R0	{1.34531, 10.6689}	{1.82851, 13.0374}	{{1.81743, 12.8385}, {1.81743, 12.8385}}	{35.9172, 22.1997}	inf	360.	1031.27	{761., 1257.}	186.463	0.446741	newly	242.	529.4	{380., 650.}	119.385	0.330138	Reff	1.0562	0.869106	{0.664816, 1.24112}	25.0907	ar	4852.	7453.6	{7252., 7658.}	53.6191
9	R0	{1.31775, 5.19226}	{2.25769, 15.3371}	{{2.2277, 15.0861}, {2.30108, 15.555}}	{71.329, 195.383}	inf	400.	1581.77	{942., 2018.}	302.385	0.623616	newly	527.	744.344	{546., 904.}	52.2288	0.231898	Reff	1.00689	1.29376	{0.767665, 1.83051}	37.9252	ar	4708.	8526.03	{8373., 8684.}	81.0967
10	R0	{1.25916, 6.89207}	{1.62191, 8.12461}	{{1.62191, 8.12461}, {1.62191, 8.12461}}	{28.8089, 17.8835}	inf	255.	443.306	{236., 734.}	77.9847	0.221884	newly	246.	340.167	{159., 633.}	53.252	0.172723	Reff	0.978871	0.725368	{0.600269, 0.949953}	29.1995	ar	3823.	6527.64	{6318., 6704.}	70.7465
11	R0	{1.20377, 5.46566}	{1.54465, 5.30296}	{{1.54465, 5.30296}, {1.54465, 5.30296}}	{28.3177, 2.97677}	inf	165.	447.286	{206., 671.}	171.082	0.397148	newly	221.	517.429	{223., 848.}	135.585	0.329471	Reff	0.992749	0.768827	{0.647826, 0.983324}	22.8369	ar	3114.	6120.93	{5888., 6326.}	96.5616
12	R0	{1.27878, 2.55804}	{2.85296, 15.4965}	{{2.86118, 15.5722}, {2.86118, 15.5722}}	{123.1, 505.796}	inf	209.	2372.07	{1093., 2843.}	1034.96	1.01407	newly	522.	1111.97	{666., 1303.}	120.036	0.34623	Reff	0.948983	1.38269	{0.827739, 2.3187}	53.2062	ar	3930.	9303.11	{9253., 9350.}	136.72
13	R0	{1.41227, 5.45696}	{2.30371, 19.6625}	{{2.30371, 19.6625}, {2.30371, 19.6625}}	{63.1211, 260.32}	inf	402.	1735.84	{1183., 2082.}	338.747	0.669375	newly	505.	627.309	{485., 745.}	33.3645	0.180149	Reff	1.01556	1.2145	{0.769439, 1.7522}	31.6335	ar	4822.	8636.65	{8547., 8714.}	79.1094
14	R0	{1.32917, 7.27593}	{2.2156, 17.3208}	{{2.15611, 16.1983}, {2.23964, 17.6755}}	{66.6903, 138.057}	inf	374.	1610.59	{1202., 1947.}	333.949	0.632204	newly	351.	645.937	{475., 774.}	89.8973	0.288561	Reff	0.994485	1.00617	{0.624188, 1.52659}	28.6519	ar	4758.	8456.22	{8284., 8612.}	77.7264
15	R0	{1.31629, 9.97481}	{1.67326, 9.40005}	{{1.67732, 9.44073}, {1.67732, 9.44073}}	{27.1192, 5.76211}	inf	325.	722.75	{458., 934.}	122.385	0.333343	newly	238.	500.075	{297., 671.}	110.662	0.307845	Reff	1.102	0.801695	{0.626026, 0.97251}	28.8893	ar	4543.	6794.83	{6573., 6961.}	49.5669
16	R0	{1.36735, 5.74166}	{2.15043, 13.9578}	{{2.08003, 13.1443}, {2.2277, 15.0861}}	{57.2696, 143.097}	inf	345.	1536.84	{1253., 1918.}	345.868	0.626482	newly	435.	774.131	{628., 921.}	84.5487	0.270847	Reff	1.05464	1.15394	{0.691425, 1.5171}	27.5279	ar	4516.	8352.07	{8171., 8553.}	84.9439
17	R0	{1.27524, 13.2396}	{1.80492, 17.6388}	{{1.70173, 15.1079}, {1.87294, 19.0269}}	{41.536, 33.4053}	inf	189.	487.239	{61., 1044.}	186.899	0.471196	newly	111.	209.457	{29., 395.}	122.503	0.413303	Reff	1.19643	1.57686	{1.32323, 1.83342}	32.6803	ar	3731.	7323.74	{6627., 7729.}	96.2943
18	R0	{1.21884, 7.71545}	{1.57759, 9.0372}	{{1.56097, 8.77207}, {1.59682, 9.30931}}	{29.4339, 17.1312}	inf	231.	583.638	{406., 802.}	152.657	0.389243	newly	192.	428.66	{285., 603.}	123.992	0.334941	Reff	1.02078	0.847837	{0.692701, 1.10158}	21.4309	ar	3080.	6307.57	{5987., 6677.}	104.791
19	R0	{1.34879, 6.71464}	{2.01161, 10.5338}	{{1.95791, 9.82151}, {2.02235, 10.6804}}	{49.1415, 56.878}	inf	378.	1118.78	{695., 1523.}	195.974	0.454816	newly	370.	675.74	{410., 917.}	84.5784	0.250691	Reff	0.926888	0.730335	{0.503379, 1.09247}	31.4575	ar	4585.	8014.64	{7846., 8141.}	74.8013
20	R0	{1.37284, 5.75036}	{1.75445, 7.55154}	{{1.75445, 7.55154}, {1.75445, 7.55154}}	{27.7971, 31.3229}	inf	291.	705.439	{477., 958.}	142.419	0.364772	newly	356.	582.707	{388., 867.}	67.6007	0.208707	Reff	1.0498	0.73639	{0.59125, 0.898454}	34.0158	ar	4750.	7167.	{7053., 7293.}	50.8842
21	R0	{1.29938, 5.36419}	{2.1456, 12.6091}	{{2.0968, 12.1899}, {2.21449, 13.2892}}	{65.1252, 135.061}	inf	347.	1491.31	{829., 1906.}	331.803	0.616995	newly	456.	814.271	{492., 995.}	83.2293	0.263744	Reff	0.968298	1.09325	{0.695928, 1.72546}	31.6057	ar	4337.	8327.42	{8113., 8551.}	92.0088
22	R0	{1.31882, 7.47626}	{1.93921, 13.9745}	{{1.86094, 12.6353}, {2.01238, 15.0173}}	{47.0414, 86.9182}	inf	278.	1284.02	{916., 1549.}	361.878	0.652722	newly	256.	641.17	{475., 745.}	150.457	0.389506	Reff	1.04292	1.02504	{0.727815, 1.46439}	21.0494	ar	3977.	7812.11	{7537., 8044.}	96.4321
23	R0	{1.26215, 3.46375}	{1.95785, 9.82221}	{{1.95791, 9.82151}, {1.95791, 9.82151}}	{55.1199, 183.572}	inf	212.	1237.5	{850., 1482.}	488.033	0.762945	newly	394.	847.043	{609., 1030.}	121.441	0.358734	Reff	0.984098	0.958671	{0.663731, 1.26207}	24.0901	ar	3607.	7854.61	{7730., 7999.}	117.76
24	R0	{1.16712, 4.19464}	{1.62648, 8.1585}	{{1.62191, 8.12461}, {1.62191, 8.12461}}	{39.3587, 94.4983}	inf	126.	432.194	{236., 712.}	243.011	0.500363	newly	188.	330.778	{159., 563.}	81.2648	0.227548	Reff	0.946418	0.719477	{0.600269, 0.949953}	27.9864	ar	3000.	6536.	{6318., 6787.}	117.867
25	R0	{1.22751, 3.34517}	{2.2909, 12.0334}	{{1.95355, 8.88568}, {2.6365, 15.4074}}	{86.6301, 259.725}	inf	170.	1207.6	{128., 2000.}	620.344	0.792494	newly	366.	789.323	{118., 1153.}	138.537	0.425521	Reff	1.07714	1.65152	{1.06506, 2.33763}	55.7645	ar	3086.	8512.63	{7898., 9208.}	175.847
26	R0	{1.18747, 5.88083}	{1.76659, 10.896}	{{1.76659, 10.896}, {1.76659, 10.896}}	{48.7692, 85.28}	inf	219.	750.711	{477., 1036.}	242.79	0.516006	newly	315.	445.579	{286., 677.}	74.9238	0.225879	Reff	0.891909	0.731043	{0.581561, 1.00413}	25.3242	ar	3614.	7210.05	{7006., 7377.}	99.5034
27	R0	{1.29023, 4.76202}	{2.43976, 19.0631}	{{2.43839, 19.0531}, {2.43839, 19.0531}}	{89.0949, 300.315}	inf	314.	1742.43	{963., 2224.}	464.979	0.747356	newly	412.	655.967	{411., 811.}	73.6003	0.293638	Reff	0.948061	1.33164	{0.736136, 1.97363}	51.5711	ar	4245.	8840.15	{8751., 8904.}	108.249
28	R0	{1.2258, 12.9056}	{1.64129, 19.8336}	{{1.6411, 19.8325}, {1.6411, 19.8325}}	{33.8953, 53.6825}	inf	204.	566.081	{266., 875.}	180.167	0.413421	newly	111.	195.946	{93., 304.}	86.6082	0.257197	Reff	0.977943	0.765564	{0.603107, 1.02569}	24.9141	ar	3639.	6652.76	{6451., 6871.}	82.8183
29	R0	{1.34702, 6.29049}	{1.98749, 11.8703}	{{1.95639, 11.2959}, {1.99822, 12.2033}}	{47.5474, 88.7027}	inf	319.	1264.61	{959., 1566.}	296.43	0.588179	newly	360.	710.352	{505., 886.}	97.32	0.285072	Reff	0.99693	0.913063	{0.635631, 1.37218}	27.3082	ar	4560.	7956.78	{7769., 8144.}	74.4907
30	R0	{1.37931, 6.78741}	{1.90884, 10.9389}	{{1.88025, 10.6367}, {1.92538, 10.9611}}	{38.3908, 61.1647}	inf	364.	793.362	{433., 1289.}	118.436	0.306926	newly	361.	461.851	{235., 822.}	49.1896	0.174546	Reff	0.975724	0.64277	{0.489239, 0.879649}	36.7899	ar	4667.	7719.85	{7577., 7882.}	65.4136
31	R0	{1.16062, 4.47529}	{1.68079, 5.99488}	{{1.59034, 5.09935}, {1.69756, 6.15907}}	{44.8186, 33.955}	inf	237.	779.222	{430., 1029.}	228.786	0.497742	newly	360.	900.267	{612., 1162.}	150.074	0.386348	Reff	0.985599	1.04057	{0.733624, 1.42701}	22.1528	ar	3037.	6828.38	{6281., 7053.}	124.84
32	R0	{1.37647, 18.7122}	{1.63728, 17.5966}	{{1.63728, 17.5966}, {1.63728, 17.5966}}	{18.9477, 5.96189}	inf	357.	465.947	{225., 740.}	48.4594	0.17424	newly	145.	178.553	{81., 280.}	46.0799	0.175317	Reff	1.02478	0.705461	{0.596461, 0.862683}	32.3287	ar	4824.	6606.68	{6401., 6777.}	36.9545
33	R0	{1.35084, 6.08841}	{2.46198, 18.8456}	{{2.46477, 18.9032}, {2.46477, 18.9032}}	{82.2556, 209.533}	inf	485.	1735.27	{477., 2313.}	267.292	0.568203	newly	533.	669.933	{228., 881.}	45.2283	0.219195	Reff	0.972335	1.44697	{0.806226, 2.18698}	55.1401	ar	4759.	8883.25	{8795., 8947.}	86.6621
34	R0	{1.36912, 6.316}	{1.81602, 10.5032}	{{1.81647, 10.5184}, {1.81647, 10.5184}}	{32.6416, 66.2952}	inf	294.	983.024	{754., 1188.}	234.362	0.513857	newly	335.	616.833	{461., 775.}	85.0533	0.257582	Reff	1.06983	0.833947	{0.65066, 1.05482}	27.0466	ar	4537.	7383.81	{7226., 7520.}	62.7465
35	R0	{1.35831, 7.09038}	{2.17428, 13.549}	{{2.11132, 12.644}, {2.21411, 14.2212}}	{60.0726, 91.0896}	inf	375.	1404.67	{829., 1855.}	274.578	0.554183	newly	358.	672.302	{381., 900.}	89.6825	0.261499	Reff	0.949459	0.748995	{0.487715, 0.996664}	30.5982	ar	4667.	8392.51	{8235., 8525.}	79.8266
36	R0	{1.28315, 5.89938}	{2.02179, 10.676}	{{2.02235, 10.6804}, {2.02235, 10.6804}}	{57.5648, 80.9683}	inf	365.	1304.65	{897., 1604.}	257.439	0.543951	newly	398.	816.224	{586., 1042.}	105.082	0.30166	Reff	0.92297	0.882805	{0.572527, 1.36549}	25.7313	ar	4460.	8031.9	{7867., 8152.}	80.0874
37	R0	{1.19303, 2.75113}	{1.69756, 6.15907}	{{1.69756, 6.15907}, {1.69756, 6.15907}}	{42.2898, 123.874}	inf	171.	764.775	{492., 993.}	347.237	0.635656	newly	357.	812.05	{472., 1068.}	127.465	0.3371	Reff	0.971365	0.892552	{0.633529, 1.28896}	23.3201	ar	3142.	6932.93	{6787., 7053.}	120.653
38	R0	{1.36033, 7.8088}	{1.87553, 9.89959}	{{1.86868, 9.75343}, {1.92538, 10.6367}}	{37.8732, 26.7748}	inf	377.	882.259	{535., 1229.}	134.423	0.350999	newly	348.	564.103	{332., 865.}	65.8343	0.203925	Reff	1.02419	0.726462	{0.546589, 1.05646}	34.3618	ar	4941.	7587.41	{7463., 7702.}	53.5603
39	R0	{1.27353, 3.70845}	{2.0968, 12.1899}	{{2.0968, 12.1899}, {2.0968, 12.1899}}	{64.6447, 228.706}	inf	255.	1435.13	{967., 1707.}	462.798	0.73949	newly	460.	822.635	{635., 977.}	79.051	0.246522	Reff	0.944322	1.09134	{0.695928, 1.68667}	31.7445	ar	3725.	8222.62	{8121., 8356.}	120.741
40	R0	{1.19983, 12.1486}	{1.59438, 19.1149}	{{1.59433, 19.1171}, {1.59433, 19.1171}}	{32.8839, 57.3427}	inf	185.	557.387	{388., 728.}	269.131	0.546566	newly	79.	199.194	{139., 264.}	152.225	0.381765	Reff	0.976302	0.840357	{0.658458, 1.05959}	22.6407	ar	3619.	6407.68	{6191., 6603.}	77.0566
41	R0	{1.20935, 2.67169}	{2.38018, 11.0886}	{{2.38758, 11.1331}, {2.38758, 11.1331}}	{96.8146, 315.042}	inf	236.	1663.47	{673., 2192.}	608.817	0.822923	newly	573.	1097.07	{618., 1372.}	101.473	0.328198	Reff	0.945228	1.37091	{0.745641, 2.09367}	53.2585	ar	3429.	8755.07	{8689., 8839.}	155.324
42	R0	{1.23313, 4.74781}	{1.67732, 9.44073}	{{1.67732, 9.44073}, {1.67732, 9.44073}}	{36.0213, 98.8439}	inf	192.	622.842	{348., 884.}	224.397	0.488957	newly	278.	422.132	{235., 632.}	58.6236	0.188256	Reff	0.975036	0.7375	{0.606016, 0.915817}	26.6596	ar	3835.	6805.08	{6638., 6961.}	77.4466
43	R0	{1.26105, 2.39713}	{2.51959, 14.0982}	{{2.41896, 13.1237}, {2.59564, 14.6975}}	{99.8008, 488.127}	inf	151.	2036.09	{1323., 2440.}	1248.4	1.11334	newly	396.	1026.39	{686., 1187.}	159.504	0.406084	Reff	0.95865	1.2183	{0.737783, 1.9687}	39.4094	ar	3723.	8952.19	{8805., 9214.}	140.456
44	R0	{1.36948, 8.88006}	{1.81513, 10.4728}	{{1.81647, 10.5184}, {1.81647, 10.5184}}	{32.5414, 17.9363}	inf	376.	1059.13	{719., 1262.}	181.684	0.438719	newly	309.	685.4	{499., 810.}	121.985	0.337092	Reff	1.06984	0.952696	{0.726043, 1.26553}	20.8917	ar	4437.	7380.4	{7222., 7520.}	66.3376
45	R0	{1.34882, 3.3791}	{2.82628, 13.2187}	{{2.52844, 11.113}, {2.95209, 14.037}}	{109.537, 291.191}	inf	365.	2138.83	{984., 2914.}	490.775	0.748544	newly	717.	1224.19	{717., 1477.}	80.5962	0.282068	Reff	1.04641	1.59216	{0.910905, 2.28293}	58.411	ar	4422.	9269.45	{9018., 9405.}	109.621
46	R0	{1.23774, 4.34917}	{1.73781, 7.10349}	{{1.7356, 7.10024}, {1.739, 7.10524}}	{40.4019, 63.3298}	inf	261.	896.87	{669., 1094.}	245.76	0.531236	newly	370.	837.152	{551., 1066.}	128.643	0.351084	Reff	0.996752	0.925157	{0.668646, 1.22286}	20.9788	ar	3685.	7101.74	{6974., 7222.}	92.7202
47	R0	{1.34327, 4.78811}	{2.79466, 18.4845}	{{2.73737, 17.9172}, {2.93978, 19.9817}}	{108.049, 286.05}	inf	281.	1676.32	{401., 2647.}	504.972	0.730499	newly	448.	707.838	{201., 1042.}	79.2312	0.295251	Reff	1.0953	1.90636	{1.14034, 2.65286}	75.0269	ar	4517.	9253.46	{9159., 9382.}	104.858
48	R0	{1.24366, 4.25814}	{2.29447, 16.0179}	{{2.2277, 15.0861}, {2.35866, 16.9062}}	{84.4934, 276.172}	inf	209.	1645.21	{955., 2045.}	688.802	0.863057	newly	310.	744.468	{509., 901.}	147.3	0.396914	Reff	0.987217	1.33737	{0.857137, 1.81803}	41.8465	ar	3678.	8619.73	{8441., 8787.}	134.359
49	R0	{1.36017, 7.5415}	{1.56097, 8.77207}	{{1.56097, 8.77207}, {1.56097, 8.77207}}	{14.7629, 16.3173}	inf	190.	501.31	{227., 746.}	163.848	0.391091	newly	183.	376.586	{166., 582.}	108.724	0.290863	Reff	1.12513	0.797984	{0.624544, 1.01838}	30.4802	ar	4178.	6247.34	{5944., 6552.}	49.5296
50	R0	{1.25333, 6.63578}	{1.67546, 9.3817}	{{1.67732, 9.44073}, {1.67732, 9.44073}}	{33.681, 41.3806}	inf	193.	507.842	{244., 759.}	163.54	0.386999	newly	204.	338.842	{151., 517.}	75.0258	0.226831	Reff	0.945763	0.687369	{0.585049, 0.833293}	28.8408	ar	3401.	6788.47	{6573., 6961.}	99.6023
